# Integration of Metabolomic and Clinical Data Improves the Prediction of Intensive Care Unit Length of Stay Following Major Traumatic Injury

**DOI:** 10.3390/metabo12010029

**Published:** 2021-12-31

**Authors:** Animesh Acharjee, Jon Hazeldine, Alina Bazarova, Lavanya Deenadayalu, Jinkang Zhang, Conor Bentley, Dominic Russ, Janet M. Lord, Georgios V. Gkoutos, Stephen P. Young, Mark A. Foster

**Affiliations:** 1 Microbiology Research Centre, National Institute for Health Research Surgical Reconstruction, Queen Elizabeth Hospital Birmingham, Birmingham B15 2GW, UK; j.hazeldine@bham.ac.uk (J.H.); conor.bentley@uhb.nhs.uk (C.B.); j.m.lord@bham.ac.uk (J.M.L.); g.gkoutos@bham.ac.uk (G.V.G.); m.foster@bham.ac.uk (M.A.F.); 2 Institute of Cancer and Genomic Sciences, University of Birmingham, Birmingham B15 2TT, UK; albazarova@gmail.com (A.B.); lavanyadeenadayalu@gmail.com (L.D.); drr719@student.bham.ac.uk (D.R.); 3 MRC Health Data Research UK (HDR UK), Midlands Site, Birmingham B15 2TT, UK; 4 Institute of Inflammation and Ageing, University of Birmingham, Birmingham B15 2TT, UK; jinkang.zhang@phe.gov.uk (J.Z.); s.p.young@bham.ac.uk (S.P.Y.); 5 National Institute for Health Research Birmingham Biomedical Research Centre, University Hospital Birmingham NHS Foundation Trust, University of Birmingham, Birmingham B15 2GW, UK; 6 Royal Centre for Defence Medicine, University Hospital Birmingham, Birmingham B15 2TT, UK

**Keywords:** metabolomics, omics integration, ICU length of stay, inflammation

## Abstract

Recent advances in emergency medicine and the co-ordinated delivery of trauma care mean more critically-injured patients now reach the hospital alive and survive life-saving operations. Indeed, between 2008 and 2017, the odds of surviving a major traumatic injury in the UK increased by nineteen percent. However, the improved survival rates of severely-injured patients have placed an increased burden on the healthcare system, with major trauma a common cause of intensive care unit (ICU) admissions that last ≥10 days. Improved understanding of the factors influencing patient outcomes is now urgently needed. We investigated the serum metabolomic profile of fifty-five major trauma patients across three post-injury phases: acute (days 0–4), intermediate (days 5–14) and late (days 15–112). Using ICU length of stay (LOS) as a clinical outcome, we aimed to determine whether the serum metabolome measured at days 0–4 post-injury for patients with an extended (≥10 days) ICU LOS differed from that of patients with a short (<10 days) ICU LOS. In addition, we investigated whether combining metabolomic profiles with clinical scoring systems would generate a variable that would identify patients with an extended ICU LOS with a greater degree of accuracy than models built on either variable alone. The number of metabolites unique to and shared across each time segment varied across acute, intermediate and late segments. A one-way ANOVA revealed the most variation in metabolite levels across the different time-points was for the metabolites lactate, glucose, anserine and 3-hydroxybutyrate. A total of eleven features were selected to differentiate between <10 days ICU LOS vs. >10 days ICU LOS. New Injury Severity Score (NISS), testosterone, and the metabolites cadaverine, urea, isoleucine, acetoacetate, dimethyl sulfone, syringate, creatinine, xylitol, and acetone form the integrated biomarker set. Using metabolic enrichment analysis, we found valine, leucine and isoleucine biosynthesis, glutathione metabolism, and glycine, serine and threonine metabolism were the top three pathways differentiating ICU LOS with a *p* < 0.05. A combined model of NISS and testosterone and all nine selected metabolites achieved an AUROC of 0.824. Differences exist in the serum metabolome of major trauma patients who subsequently experience a short or prolonged ICU LOS in the acute post-injury setting. Combining metabolomic data with anatomical scoring systems allowed us to discriminate between these two groups with a greater degree of accuracy than that of either variable alone.

## 1. Introduction

Recent advancements in emergency medicine and the co-ordinated delivery of trauma care mean more critically-injured patients now reach the hospital alive and survive life-saving operations. Indeed, between 2008 and 2017, the odds of surviving a major traumatic injury in the UK increased by nineteen percent [1]. However, the improved survival rates of severely-injured patients have placed an increased burden on the healthcare system, with major trauma a common cause of intensive care unit (ICU) admissions that last ≥14 days [2,3,4]. Despite representing a small proportion of all hospital admissions, critically-ill patients with a prolonged ICU stay consume the greatest amount of resources on ICU wards [5,6,7]. Moreover, patients who experience an extended ICU length of stay (LOS) develop more secondary complications [6,8], are at a higher risk of in-hospital mortality [9] and are frequently discharged to rehabilitation centres or skilled nursing facilities [6]. Thus, to allocate resources and improve clinical outcomes, there is an urgent need to develop strategies to identify trauma patients at risk of a prolonged ICU LOS at the earliest opportunity.

Across the settings of traumatic brain injury (TBI), blunt trauma and penetrative injury, prospective and retrospective observational studies have identified several demographic and clinical factors influencing ICU LOS. Falling into one of three distinct categories that relate to (i) pre-trauma parameters, (ii) the traumatic injury itself, or (iii) post-trauma treatment regimens and secondary complications, several factors have been reported to be associated with a prolonged ICU LOS. These factors include male gender [9], increased age [6,9,10], pre-existing co-morbidities [6], higher injury severity [6,9,10], severe head injury [6,10,11,12], surgical procedures [10] and nosocomial infection [9,10]. While an assessment of injury severity at hospital arrival, using either the injury severity score (ISS) or new ISS (NISS), can predict who requires admission to ICU [13,14,15,16], these anatomical scoring systems are unable to accurately distinguish between patients who subsequently experience a short or prolonged ICU LOS [17,18,19], with the area under the receiver operating characteristic (AUROC) curve values ranging from 0.448–0.804 [17,18,19]. Thus, new models capable of predicting ICU LOS with greater discriminatory power are needed to aid in the development of targeted treatments during the acute post-injury phase.

Metabolomics is a systems-based approach for analysing low molecular weight metabolites present in cells, tissues or biofluids. Applying this analytical technique to serum or plasma samples acquired in the minutes [20], hour [21,22,23] and days [23,24,25,26] following combat injury, TBI or blunt/penetrative trauma has revealed significant trauma-induced metabolic perturbations, with changes reported in glucose, fatty acid, amino acid and lipid metabolism. Showing promise as a diagnostic tool for mild TBI [27] and stratifying patients according to injury severity [22,23], metabolic profiling of the circulating metabolome has revealed unique signatures for patients with distinct clinical outcomes. For example, disturbances in amino acid and fatty acid metabolism have been reported in TBI patients who experience post-traumatic cognitive impairments [21], whilst significantly increased concentrations of glucose and glutamate were measured in serum samples obtained at the time of hospital admission from non-survivors of severe trauma [20]. More recently, in a cohort of eighty-six blunt trauma patients, Cyr et al. found that at days 2–5 post-injury, a plasma metabolome enriched in sphingolipids was associated with several improved clinical outcomes, which included a shorter ICU LOS [28]. However, the study did not address whether metabolomic profiles in the immediate post-injury phase could be used to predict which patients would experience an extended LOS in ICU. 

Data are emerging to suggest that metabolomic profiling can markedly improve the accuracy of statistical models built on clinical parameters to predict patient outcomes. For example, Oresic and colleagues found that combining data on serum metabolites with clinical indices generated a variable that discriminated between TBI patients who reported good and poor outcomes with a level of accuracy that was significantly greater than that of a model based on clinical information alone [22]. Integrating metabolomic and clinical data has also proven helpful in identifying critically-ill patients that are at an increased risk of mortality, with this approach outperforming the predictive abilities of such widely used clinical scoring systems as the sequential organ failure assessment (SOFA) score and the acute physiology and chronic health evaluation II (APACHE II) score [29]. Thus, combining metabolomic profiles with clinical data could be one strategy to improve anatomical-based scoring systems’ ability to identify patients at risk of a prolonged ICU LOS.

Here, in this prospective observational study, we investigated the serum metabolomic profile of fifty-five major trauma patients across three post-injury phases: acute (days 0–4), intermediate (days 5–14) and late (days 15–112). Using ICU LOS as a clinical outcome, we aimed to determine whether the serum metabolome measured at days 0–4 post-injury for patients with an extended (≥10 days) ICU LOS differed from that of patients with a short (<10 days) ICU LOS. In addition, we investigated whether combining metabolomic profiles with clinical scoring systems would generate a variable that would identify patients with an extended ICU LOS with a greater degree of accuracy than models built on either variable alone. 

## 2. Methods

### 2.1. Study Design 

This study presents data obtained from subjects enrolled into the Steroids and Immunity from Injury through to Rehabilitation (SIR) study, a prospective cohort study conducted between May 2011 and January 2013 in the Royal Centre for Defence Medicine and Queen Elizabeth Hospital Birmingham, UK. The study protocol was approved by the National Research Ethics Committee South West-Frenchay (Reference-11/SW/0177) and the Ministry of Defence Research Ethics Committee (Reference-116/Gen/10). Details of study inclusion/exclusion criteria and the consenting procedure have been described previously [30].

### 2.2. Clinical Data Collection

Simplified Acute Physiology Score (SAPS), APACHE II, and SOFA scores were calculated daily from the ICU clinical charts while admitted to ICU. Data regarding mortality, ICU and hospital LOS, ISS, NISS and abbreviated injury scale scores were calculated and ratified using the hospital Prescribing Information and Communication System (PICS, University Hospitals Birmingham Foundation Trust), the Trauma Audit Research Network (TARN), a UK-based centralised network that records injury details, or the UK Military equivalent, the Joint Theatre Trauma Registry (JTTR). 

### 2.3. Clinical Outcomes

The clinical course of severely injured trauma patients is complicated by persistent inflammation, immunosuppression, and catabolism syndrome (PICS). Patients with an ICU LOS greater than 10 days are more likely to develop PICS that can progress to multiple organ dysfunction syndrome and multiple organ failure [31]. Thus, in this study, we categorised ICU LOS as short (<10 days) or prolonged (≥10 days). We provided clinical data in the supplementary Table S1.

### 2.4. Blood Sampling and Preparation of Serum

Blood samples were collected within 24 h of injury and between 07:30 and 09:00 on days 3, 5, 10, 14, 21, 28, and months 2, 3, 4 and 6 post-injury. Samples were collected in BD Vacutainers^®^ (BD Biosciences, Plymouth, UK) containing z-serum clotting activator. After a 30-min incubation at room temperature (RT), samples were centrifuged at 1620× *g* for 10 min at RT. Serum was harvested and stored at −80 °C until analysed.

Comparison of ICU LOS < 10 days vs. ≥10 days. Unless stated, data are expressed as means with ranges in parentheses. APACHE, Acute physiology chronic health evaluation; GCS, Glasgow coma scale; GSW, Gunshot wound; ICU, Intensive care unit; IED, Improvised explosive device; ISS, Injury severity score; LOS, Length of stay; NISS, New injury severity score; RTC, Road traffic collision; RTS, Revised trauma score; SAPS, Simplified acute physiology score; SOFA, Sequential organ failure assessment score; TRISS, Trauma and injury severity score.

### 2.5. Metabolomics Sample Preparation and Analysis

To remove proteins, which reduce the quality of the NMR spectra, serum was filtered through 3000 MWCO filters [32]. The filtrates were pH buffered with 50 mM phosphate and made to 10% with D_2_O and 0.5 mM TMSP (an internal NMR standard). We then assessed the metabolomic profile using NMR spectroscopy as previously described [33,34].

1D 1H NOESY NMR spectra were acquired at 300 K using a standard 1D-1H-Nuclear Overhauser Effect Spectroscopy pulse sequence with water saturation using pre-sat in a Bruker AVANCE II 600 MHz NMR spectrometer (Bruker Corp., Billerica, MA, USA) equipped with a 1.7 mm cryoprobe. Spectral width was set to 12 ppm, and the scans were repeated 128 times. Spectra were processed using Metabolab within MATLAB (Version 9.2, Mathworks, Cambridge, UK) [35]. Using this package, spectra were phased manually, baseline corrected and referenced to the TMSP resonance (0.0 ppm). To facilitate analysis, each spectrum between 0.2 and 9.0 ppm was reduced in complexity by segmenting into 0.005 ppm bins (2.5 Hz). Bins between 4.5 and 5.0 ppm containing residual water were removed, and the remaining bins were integrated. Each binned spectrum was normalised using probabilistic quotient normalization [36], transformed using a generalised logarithm (glog) [37] to increase the weighting of smaller peaks. Minor batch anomalies were corrected using the Combat algorithm with the SVA package [38]. Metabolites were identified by comparing spectra with spectra in the human metabolome database [39], using the Chenomx suite of software containing a metabolite library (Chenomx Inc, Edmonton, AB, Canada). Metabolic pathways involved or changes in different subgroups were identified and portrayed using the Metaboanalyst (http://www.metaboanalyst.ca/MetaboAnalyst, accessed on 1 November 2021) version 4.0 software [40].

All metabolites or inflammatory mediators with more than 50% missing values were excluded from the analysis. Data pre-processing includes logarithmic transformation, centring, and unit variance scaling) was first conducted separately for the metabolomics and other clinical variables and then combined dataset. We provided processed metabolomics data in the supplementary Table S2. 

### 2.6. Statistical Analysis

We used univariate ANOVA analysis with post hoc correction (*p* < 0.05) to identify the number of metabolite peaks that were significantly different across different time points. To integrate these with the number of ICU days, we used simple linear regression with a false discovery rate correction (FDR) method (Benjamini–Hochberg Procedure (BH) correction) at *p* < 0.05. 

### 2.7. Partial Least-Squares Discriminant Analysis (PLS-DA) Analysis 

For dichotomous or binary outcomes (ICU LOS < 10 vs. ≥10 days), we used a supervised classification method called partial least-squares discriminant analysis (PLS-DA). We quantified model statistics based on the fraction of the sum of squares for the selected component (R2), which equates to the percentage of the model variance explained, and the predictive ability (Q2). A 10 fold cross-validation was performed to predict and estimate the model performance (whether models were overfitted). In addition to this we have performed a permutation test to derive probability values from the fitted models. We used variable importance in the projection scores (VIP) to prioritize metabolites and clinical features. VIP scores of more than one are considered important, and this score of a variable is calculated as a weighted sum of the squared correlations between the PLS-DA components and the original variable. For metabolomics and clinical data, we used a VIP score of more than 1.5 as a cut-off [41]. Further, we used AUC values to provide predictive aspects of the combined markers selected. For analysis, we used the Metaboanalyst version 4.0 software [40] for both model building and VIP score estimation. 

## 3. Results

### 3.1. Patient Demographics

A total of 55 male adult trauma patients were enrolled into the study (Table 1). Improvised explosive device was the most common mechanism of injury (n = 31, 56%) in a cohort whose mean age was 29 years (range, 18–50 years). With a mean ISS of 25.5 (range, 9–75), patients were severely-injured upon hospital arrival and were critically unwell on the day of injury, as demonstrated by mean SOFA and APACHE II scores of 8.3 (range, 0–17) and 20.3 (range, 0–34) respectively (Table 1).

Patients with an ICU LOS ≥ 10 days (n = 26, 47%) were more severely-injured and critically unwell than patients with an ICU LOS < 10 days (n = 29, 53%), presenting on the day of injury with significantly higher ISS (30 (range, 13–75) vs. 21 (range, 9–38), *p* = 0.005) and APACHE II scores (22.4 (range, 7–34) vs. 17.9 (range, 0–29), *p* = 0.04) (Table 1). The length of time receiving ventilator support was significantly longer for patients with an ICU LOS ≥ 10 days (16.2 (range, 10–33) vs. 4.6 (range, 0–9) days, *p* = <0.0001) who also had a significantly greater hospital LOS (59 (17–217) vs. 34 (7–72) days, *p* = 0.02) (Table 1).

### 3.2. Changes in the Serum Metabolome over Time 

**Figure 1:**
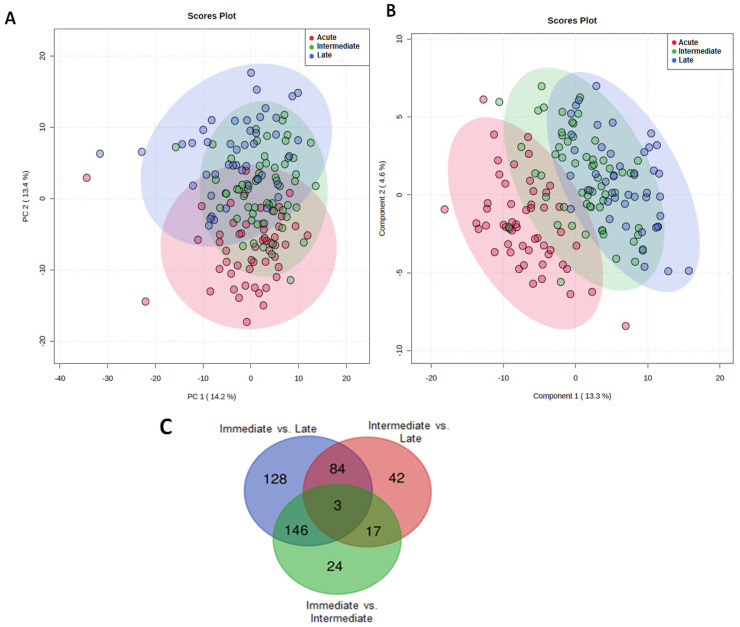

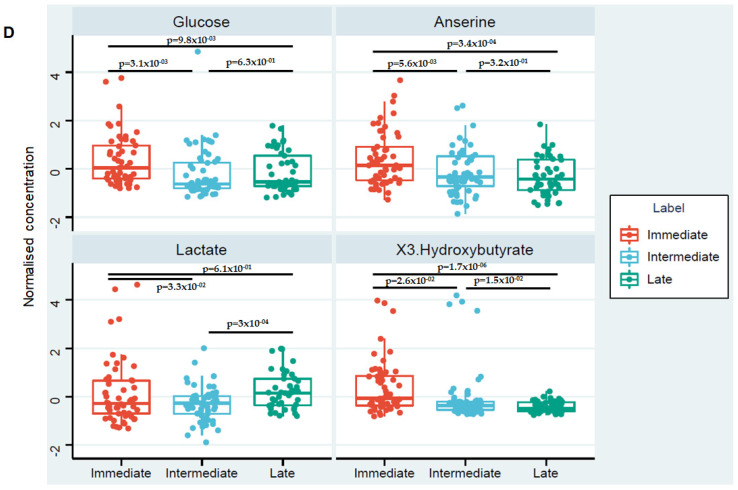
The effect of major traumatic injury on the serum metabolome over time. (**A**) Unsupervised PCA score plot (**B**) Supervised PLS-DA score plot analysis of metabolomic data when analysed according to time of sample acquisition. Data are segmented into 3 post-injury phases: acute (red dots), intermediate (green dots) and late (blue dots). Each dot represents a patient. (**C**) Number of significant metabolites unique to each of the post-injury sampling time-points and the number of common metabolites measured at each time segment. (**D**) Box plots showing the differences in the levels of metabolites putatively identified as glucose, anserine, lactate and 3-hydroxybutyrate between the three-time segments.

To understand how the metabolic response to major trauma changed over time, we first compared the serum metabolomes at each of our three post-injury sampling phases. Unsupervised PCA (Figure 1A) and supervised PLS-DA (Figure 1B) analysis demonstrated marked variation in the metabolomic profiles measured at the acute, intermediate and late post-injury time-points. 

To identify the significant metabolites in each of these segments, a one-way ANOVA was performed with at *p* < 0.05, Fisher’s LSD corrected. Figure 1C shows the number of metabolites unique to and shared across each time segment. A one-way ANOVA revealed the most variation in metabolite levels across the different time-points was for metabolites putatively identified as, glucose, anserine and 3-hydroxybutyrate respectively. The heatmap presented in the supplementary Figure S1 summarises the changes that occurred in the levels of these metabolites across the three post-injury phases. The percentage of the model variance explained (R2), and the predictive ability (Q2) were found 86% and 58% respectively. The PLS model was found statistically significant at *p* < 0.01 after permutation test (supplementary Figure S2). Compared to samples acquired in the acute injury setting, significantly lower glucose levels, anserine and 3-hydroxybutyrate were detected at the intermediate and late sampling time-points (Figure 1D). Serum levels of lactate were significantly higher in the late post-injury phase when compared to the intermediate sampling time-point (Figure 1D).

### 3.3. Trauma Patients with an ICU LOS ≥ 10 Days Exhibit a Distinct Metabolomic Profile in the Acute Injury Phase

**Figure 2:**
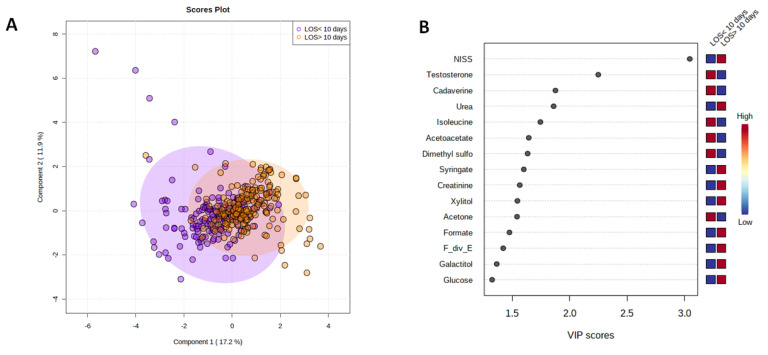

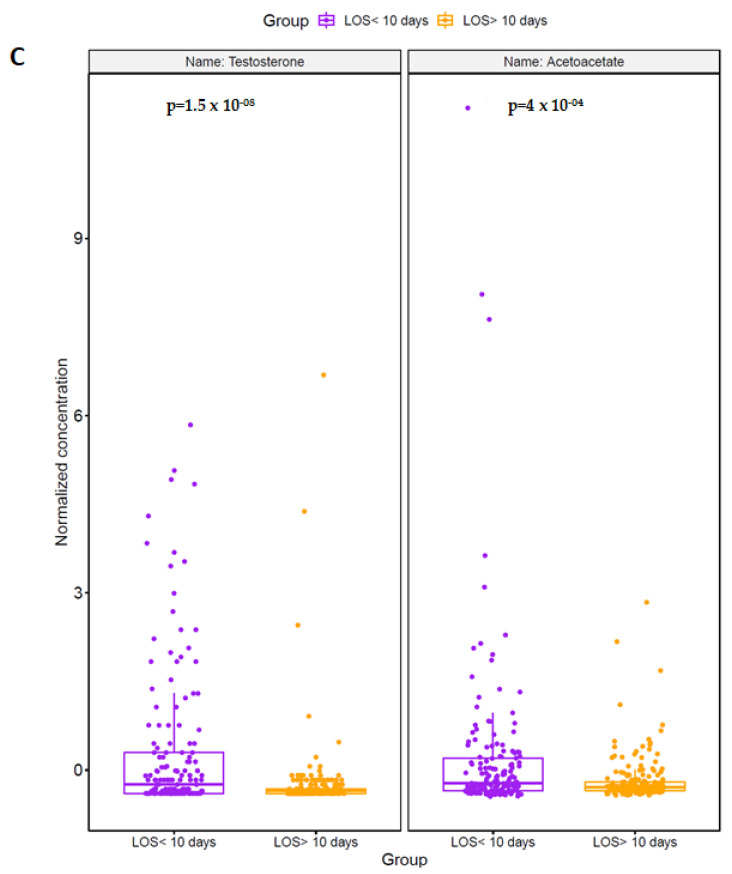
Trauma patients with an ICU LOS ≥10 days present with a distinct serum metabolome in the acute post-injury phase. (**A**) A PLS-DA score plot highlighting the differences in the serum metabolome in the acute post-injury phase of patients with an ICU LOS < 10 (purple dots) or LOS ≥ 10 days (orange dots). (**B**) VIP score plot showing the 11 selected features with a VIP score > 1.5 that discriminated between ICU patients with a LOS < 10 or ≥ 10 days. (**C**) Comparison of testosterone and acetoacetate levels in serum samples acquired in the acute post-injury phase between patients who experienced an ICU LOS < 10 or ≥10 days.

PLS-DA models were constructed to investigate whether serum metabolites or clinical variables could be used to discriminate between patients with a short or extended ICU LOS. We aimed to identify potential biomarkers of poor clinical outcome in the acute injury setting. Only metabolomic data derived from the analysis of serum samples acquired in the acute post-injury phase were studied. As shown in (Figure 2A), patients with a prolonged ICU LOS exhibited a distinct metabolomic signature. To identify candidate biomarkers for patients with an ICU LOS ≥ 10 days, features were ranked based on their Variable Importance in Projection (VIP) score and selected if they had a score of more than 1.5. A total of eleven features were selected; NISS, testosterone, and the metabolites cadaverine, urea, isoleucine, acetoacetate, dimethyl sulfone, syringate, creatinine, xylitol and acetone (Figure 2B). The percentage of the model variance explained (R2), and the predictive ability (Q2) were found 33% and 29% respectively. The PLS model was found statistically significant at *p* < 0.01 after permutation test (Supplementary Figure S3). Univariate Kruskal–Wallis analysis found that two of these features, namely testosterone and acetoacetate, were significantly different between the two groups, with their levels higher in patients with an ICU LOS < 10 days (Figure 2C). Comparing the metabolites mentioned above at the intermediate and late post-injury phases found no significant differences between the two patient groups.

### 3.4. Clinical Variables and the Acute Metabolic Response Can Discriminate between Patients with a Short or Extended ICU LOS

**Figure 3:**
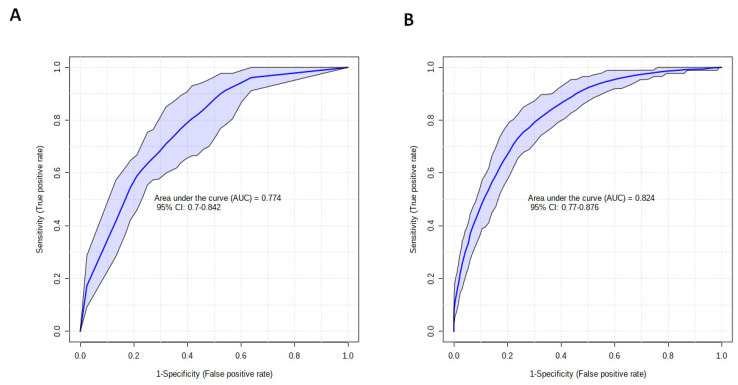
Metabolomic profiling improves the accuracy of clinical variables for discriminating between trauma patients with a short or prolonged ICU LOS. Receiver operator characteristic curves for models built on: (**A**) the combined variables of NISS and testosterone, or (**B**) NISS and all 9 serum metabolites (dimethyl sulfone, cadaverine, isoleucine, acetoacetate, urea, syringate, acetone, xylitol and creatinine) detected in the acute post-injury phase that were selected from our PLS-DA model for discriminating between patients with an ICU LOS < 10 or ≥10 days.

To determine whether injury severity, as assessed by the NISS, or metabolites, detected in the acute post-injury phase, could be of prognostic value, we examined the ability of the eleven features selected from our PLS-DA model (Figure 2B) to discriminate between patients with a short or prolonged ICU LOS. The AUROC values for each element are provided in Table 2. As single variables, NISS produced the highest AUROC value of 0.69. A combined model of NISS and testosterone, the two features that demonstrated the greatest discriminatory power as single variables, generated an AUROC of 0.77 (Figure 3A). A model constructed on all nine selected metabolites alone achieved an AUROC of 0.78, which increased to 0.824 when combined with NISS (Figure 3B). 

### 3.5. Altered Amino Acid Metabolism Is a Feature of the Acute Metabolic Response for Patients with a Prolonged ICU LOS

**Figure 4:**
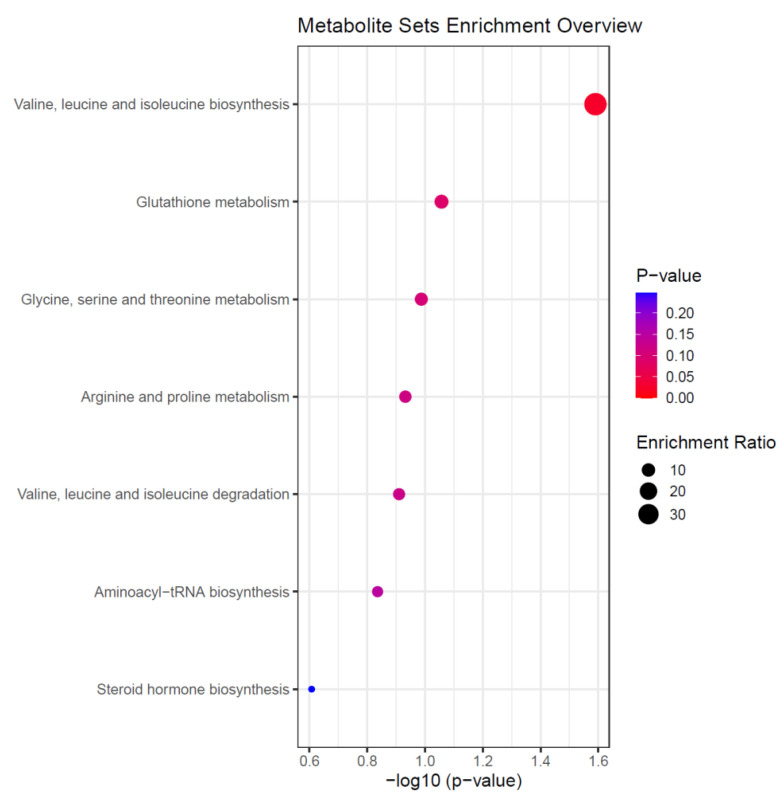
Summary of the altered metabolic pathways in patients with an ICU LOS ≥ 10 days. Data were derived from enrichment analysis of the nine selected metabolites from our PLS-DA model for discriminating between patients with an ICU LOS < 10 or ≥10 days. Enrichment Ratio is computed by Hits/Expected, where hits = observed hits; expected = expected hit.

To identify the metabolic pathways associated with the selected metabolites, and thus an ICU LOS ≥ 10 days, enrichment analysis using MetaboAnalyst was performed. We found valine, leucine and isoleucine biosynthesis, glutathione metabolism, and glycine, serine and threonine metabolism were the top three pathways with a *p* < 0.05 (Figure 4). 

## 4. Discussion 

Ranging from an increased incidence of secondary complications to discharge to specialised care facilities, critically ill patients with an extended ICU LOS experience poor short and long-term clinical outcomes [6,8]. In paediatric and adult trauma settings, several groups have investigated whether an assessment of injury severity at hospital presentation can aid in the identification of patients at risk of a prolonged ICU LOS [17,18,19,42]. Whilst some studies have reported either no or weak positive associations between injury severity scores and ICU LOS [42], others have shown that statistical models built on the anatomical scoring systems of ISS or NISS can distinguish between patients who experience a short or long ICU LOS [17,18,19]. However, the predictive ability of these models range from poor to fair, meaning there is a need to develop new models with greater discriminative power. Here, we have analysed the serum metabolome of fifty-five severely injured male trauma patients, describing significant changes across time in metabolic pathways related to carbohydrate, amino acid and fatty acid metabolism. Importantly, analysis of samples collected in the acute post-injury phase revealed distinct metabolomic profiles for patients who subsequently experienced a prolonged ICU LOS. To predict an ICU LOS ≥ 10 days, a combined anatomical-physiological model built on nine serum metabolites and NISS outperformed models designed on either variable alone. Thus, akin to findings reported in other critical care settings [29], our data demonstrate the importance of studying the physiological response to major trauma when identifying patients at risk of poor clinical outcomes.

Our current understanding of the metabolic response to traumatic injury is based almost entirely upon the results of studies that have analysed serum samples acquired from patients at either a single post-injury time-point [20,21,22,24,43] or at multiple time-points during the acute injury phase (days 1–7) [23,25,26,28,44,45]. Thus, via the analysis of blood samples obtained in the acute (days 0–4), intermediate (days 5–14) and late (days 15–112) post-injury phases, our study has provided novel insights into both the kinetics of the metabolic response to injury as well as the long-term metabolomic profiles of major trauma patients. Revealing time-associated alterations in lipid, protein and carbohydrate metabolism, we detected significantly lower levels of glucose, anserine and 3-hydroxybutyrate in serum samples acquired from patients at our intermediate and late post-injury time-points when compared to the levels present during the acute sampling phase. Major trauma patients presenting with hyperglycaemia and elevated ketone bodies at hospital admission have been reported previously [20,23]. Mechanistically, these early perturbations in carbohydrate and fatty acid metabolism are likely to be driven by immediate trauma-induced changes in the circulating hormonal milieu. For instance, catecholamines, glucocorticoids, growth hormone and/or glucagon, whose levels are all elevated post-injury, promote peripheral insulin resistance, reduce muscle glucose uptake and trigger hepatic glycogenolysis, gluconeogenesis, and ketogenesis [46,47,48,49,50]. In the only other study to our knowledge to have prospectively studied the circulating metabolome of injured military personnel, Lusczek et al. analysed serial plasma samples from seventy-eight combat causalities in the acute injury phase (0–24 h). In line with the changes we observed in carbohydrate metabolism over time, the authors detected lower glucose concentrations 8 and 24 h post-injury compared to the levels they measured in samples acquired at hospital admission [23]. However, in contrast to our observations, the same study found concentrations of 3-hydroxybutyrate increased in the circulation of major trauma patients over time [23]. This difference may reflect our different sampling times or that glucose levels were sufficiently low in their patient cohort to require an increase in ketoacidosis. 

Across time windows of 0–24 and 0–120 h, longitudinal studies of blunt trauma patients and combat casualties have reported a time associated decrease in the plasma levels of lactate [23,28]. In contrast to these observations, we found lactate levels were significantly higher in the serum samples we acquired from patients at our late stage sampling time-point when compared to the intermediate phase. Based on the findings of the above-mentioned studies, our observation of hyperlactaemia in trauma patients sampled 15–112 days post-injury is surprising. The differences observed in our study could be attributed to the high number of severely injured patients that remained on ICU with ongoing multiple organ dysfunction/failure. Early resuscitation, restoring tissue perfusion will clear lactate from the circulation and is indeed used as a surrogate measure of resuscitation success. While ongoing multi-organ dysfunction and sepsis will generate more lactate, and so it is unreliable [51,52].

In civilian and military trauma settings, distinct metabolic phenotypes have been described for patients who experience poor clinical outcomes. Adding to a body of literature that has demonstrated distinct metabolomic profiles for major trauma patients who: (i) succumb to their injuries [20,23,24,45,53] (ii) require extended ventilator use [28] or (iii) develop nosocomial infections that progress to sepsis, [28,54] we have shown that the early metabolic response to injury differs between patients who experience a short or extended ICU LOS. Analysing samples acquired in the acute post-injury phase, we detected a distinct metabolomic signature for patients with a prolonged ICU LOS, with VIP scores revealing that testosterone and nine metabolites (cadaverine, urea, isoleucine, acetoacetate, dimethyl sulfone, syringate, creatinine, xylitol and acetone) made the greatest contributions to PLS-DA models that distinguished between trauma patients with an ICU LOS < 10 or ≥10 days. Of these selected features, testosterone levels and the ketone body acetoacetate were significantly higher in patients with an ICU LOS < 10 days. 

Our observation that hypogonadism is associated with poor clinical outcomes post-trauma agrees with the results of previous prospective studies of critically ill patients that found low testosterone levels were associated with and/or predictive of mortality, increased ventilator use, a prolonged ICU LOS and cognitive/functional decline [55,56,57,58]. Although evident within hours of injury and persisting for up to two months [30], how a trauma-induced reduction in testosterone levels influences the physiological response to injury in humans is poorly understood [59]. The central stimulation of the hypothalamic pituitary adrenal axis, unhindered by the suppression of the gonadal axis, has been well reported, but how much this is due to design or consequence is unknown [59]. Given that testosterone is important for wound healing and the maintenance of bone density and muscle mass, we speculate that hypogonadism post-trauma contributes to a state of systemic catabolism and promotes poor wound healing that leads to an extended ICU LOS [60]. In support of this idea, we have previously shown that the gradual recovery in gonadal androgen production post-injury coincides with a transition from catabolism to anabolism, as assessed by urinary nitrogen excretion and biceps brachii muscle thickness [30]. Thus, could androgen supplementation be used to aid clinical recovery and reduce ICU LOS post-trauma? Widely studied in the setting of thermal injury, treatment with the synthetic androgen oxandrolone has been shown to improve donor-site healing time and reduce net weight and lean body mass loss in patients with severe burns [61]. Associated with a shorter hospital LOS, these clinical benefits highlight the potential therapeutic value of androgen supplementation [61]. That said, whether such benefits extend to major trauma patients is currently unclear. For example, whilst reduced nitrogen and/or 3-methylhistidine excretion has been reported for polytrauma and TBI patients treated with the testosterone analogue nandrolone [62,63], oxandrolone therapy in trauma patients resulted in prolonged ventilator use and a longer ICU LOS [64]. As these studies recruited a relatively small number of patients with different mechanisms of injury and varied not only in the type of nutritional support they provided to patients but also in the dose and timing of steroid therapy, further studies are needed to establish whether treatment with anabolic steroids can improve the clinical outcomes of major trauma patients. 

A product of fatty acid metabolism, ketone bodies increase cerebral blood flow and protect neuronal cells from stress and cytotoxicity-induced apoptosis [65]. However, as patients who had TBI as a significant component of their injury were excluded from our study, it is unlikely that these neuroprotective effects contributed to the association we observed between raised acetoacetate levels and a reduced ICU LOS. Aside from its cerebral benefits, acetoacetate, via activation of mitogen-activated protein kinase signalling, accelerates muscle cell proliferation and regeneration [66]. Moreover, in the mitochondrial matrix, acetoacetate can be reduced to 3-hydroxybutyrate, reducing oxidative stress and blunt pro-inflammatory cytokine responses [67]. Thus, these anti-catabolic and anti-inflammatory properties provide potential mechanistic explanations for the improved clinical outcomes we observed for patients with elevated circulating levels of acetoacetate post-injury. 

Previous studies have suggested that a patient’s metabolic response to trauma impacts upon their ICU LOS. Associated with significantly lower levels of circulating pro-inflammatory mediators, Cyr et al. found that patients who presented 2–5 days post-injury with a plasma metabolome enriched in sphingolipids had a shorter ICU LOS than patients who exhibited no time-dependent change in sphingolipid levels [28]. Conversely, increased levels of metabolites related to amino acid and glucose metabolism were measured in plasma samples acquired 12–24 h post-injury from blunt and penetrating trauma patients who experienced an ICU LOS ≥ 14 days compared to those who recovered rapidly (ICU LOS ≤ 7 days) [44]. However, no study to our knowledge had investigated whether metabolic profiles could be used to predict a patient’s ICU LOS. For discriminating between patients with an ICU LOS < 10 or ≥10 days, testosterone and the nine metabolites selected from our PLS-DA plots were generated as single variables, AUROC values lower than those derived from a model built on a patients NISS. However, a model constructed on all nine metabolites provided greater discriminatory power than NISS alone. The predictive ability of the model, Q2 was low which indicates that ICU LOS is a complex clinical measure and very few metabolites impacting the outcome variable. That model built on combinations of metabolites outperform those designed on single metabolites for predicting patient outcome has been demonstrated previously. For example, Servia and colleagues reported that combined measurement of cortisol and myristic acid discriminated between survivors and non-survivors of traumatic injury with greater accuracy than a panel of six individual metabolites [24]. With an AUROC value of 0.824, our best model for distinguishing between patients with an ICU LOS < 10 or ≥10 days was built upon combining our nine selected metabolites and a NISS and gives an 18% improvement over NISS alone. However, NISS still acts as one of the important predictors to discriminate short (<10 days) vs. long (>10 days) ICU LOS. Highlighting the benefits of studying the physiological response to injury, this observation adds to data derived from other studies of critically-ill patients where merging clinical and metabolomic data improved the accuracy of models designed to predict poor outcome following TBI22 or mortality risk in septic patients [29]. With technological advancements now making it possible to process metabolomic data within 3-h of sample analysis [68], it is conceivable that clinicians could gain access to the metabolic profiles of their patients in the acute injury phase. Our study suggests that such data, when used alongside anatomical scoring systems, could be used to assist in the design of personalised treatment and/or management protocols for patients at risk of poor clinical outcomes. 

This study has several limitations. Our findings require validation in larger independent cohorts by enrolling a relatively small number of patients into a study conducted at a single major trauma centre. Related to this, our analyses were performed on samples acquired only from male trauma patients of young to middle age. Thus, whilst beneficial in reducing the confounding effects that age and sex have on the metabolic response to traumatic injury [25], this patient demographic means our results may not be generalizable to other trauma patients. The generalisability of our findings may also be hampered by our recruitment of both civilian and military trauma patients who presented with diverse patterns and severities of injury. However, it should be noted that for our assessment of whether metabolomic profiles could be used to discriminate between patients according to their ICU LOS, no significant differences were detected in the mechanisms of injury with an ICU LOS < 10 or ≥10 days. The most important limitation of our study was the absence of a healthy control (HC) cohort, which prevented us from reporting upon both the short and long-term effects of major trauma on the serum metabolome. Previous trauma-based studies lacking a HC cohort have used metabolomic data obtained from independent studies as reference values for comparison [28]. The inclusion of military personnel within our trauma cohort prevented us from adopting this approach as military training alone results in distinct metabolomic signatures [69,70].

## 5. Conclusions

In summary, we have shown that differences exist in the serum metabolome of major trauma patients who subsequently experience a short or prolonged ICU LOS in the acute post-injury setting. Combining metabolomic data with anatomical scoring systems allowed us to discriminate between these two groups with a greater degree of accuracy than that of either variable alone. When considered alongside the results of studies that have reported distinct genomic signatures for trauma patients who develop multiple organ failure and nosocomial infections [71,72], our data highlights the benefits offered by “omics” research for aiding in the identification of patients at risk of poor clinical outcome following major traumatic injury.

## Figures and Tables

**Table 1:** Summary of the demographic and clinical characteristics of the study cohort.

Parameters	Patients (N = 55)	ICU LOS < 10 Days (N = 29)	ICU LOS ≥ 10 Days (N = 26)	*p*-Value
Age, years	29 (18–50)	27.5 (18–49)	31 (21–49.5)	0.17
Male, n (%)	55 (100)	29 (100)	26 (100)	N/A
Mechanism of Injury Explosive, n (%) GSW, n (%) Electrical, n (%) Blunt, n (%) Penetration, n (%)	31 (56.3 %) 8 (14.5 %) 1 (1.8 %) 13 (23.6%) 2 (3.6 %)	16 (55.2%) 5 (17.2 %) 0 7 (24.1%) 1 (3.5%)	15 (57.7%) 3 (11.5%) 1 (3.8 %) 6 (23.1 %) 1 (3.9%)	NA
ISS	25.5 (9–75)	21 (9–38)	30 (13–75)	0.005
NISS	37.3 (16–75)	34.4 (17–59)	40.6 (16–75)	0.07
GCS	10.3 (3–15)	10.7 (3–15)	9.9 (3–15)	0.61
SOFA (Day 1)	8.3 (0–17)	15 (7.72–17)	8.6 (0–15)	0.41
APACHE II (Day 1)	20.3 (0–34)	17.9 (0–29)	22.4 (7–34)	0.04
SAPS II (Day 1)	42.2 (0–69)	38.6 (11–62)	44.7 (0–69)	0.21
TRISS (Day 1)	77.13 (2.55–99.4)	78.23 (2.55–99.4)	75.9 (13.27–98.6)	0.76
RTS (Day 1)	6.3 (2.6–7.8)	6.4 (2.91–7.84)	6.1 (2.63–7.84)	0.55
Ventilator days	7.8 (0–25)	3.3 (0–10)	12.9 (4–25)	<0.0001
Operative procedures	5.8 (0–24)	5 (0–15)	6.8 (0–24)	0.12
ICU LOS	10 (0–34)	4.6 (0–9)	16.2 (10–33)	<0.0001
Hospital LOS	46 (7–217)	34 (7–72)	59 (17–217)	0.02

**Table 2:** Area under the receiver operating characteristic curve (AUROC) values, confidence intervals (C.I) and p values for the metabolites and clinical variables selected from our PLS-DA model based on a VIP > 1.5.

Feature	AUROC	Confidence Interval	*p* Value
NISS	0.69434	0.639–0.748	2.62 × 10^−12^
Testosterone	0.668566	0.612–0.728	3.27 × 10^−7^
Dimethyl sulfone	0.636886	0.577–0.694	0.000234
Cadaverine	0.628626	0.55–0.682	2.25 × 10^−5^
Isoleucine	0.625872	0.568–0.68	8.30 × 10^−5^
Acetoacetate	0.609176	0.558–0.675	0.000214
Urea	0.595153	0.536–0.652	2.64 × 10^−5^
Syringate	0.591759	0.537–0.645	0.000314
Acetone	0.589022	0.434–0.621	0.000523
Xylitol	0.583723	0.522–0.64	0.000509
Creatinine	0.552331	0.512-0.612	0.000426

## Data Availability

Data can be found in the supplementary files or https://figshare.com/account/home#/projects/129278.

## Supplementary Materials

The following supporting information can be downloaded at: www.mdpi.com/article/10.3390/metabo12010029/s1, Figure S1: Heatmap of the selected metabolites across multiple patients; Figure S2: Permutation test statistics over 100 iterations across multiple segments; Figure S3: Permutation test statistics over 100 iterations with selected metabolites for ICU LOS > 10 vs. <10; Table S1: List of the clinical parameters; Table S2: List of the selected metabolite data set.
