# Supplementary material for: Integration of Metabolomic and Clinical Data Improves the Prediction of Intensive Care Unit Length of Stay Following Major Traumatic Injury
Source: Metabolites. 2021 Dec 31;12(1):29. doi: 10.3390/metabo12010029 (PMC8780653; doi:10.3390/metabo12010029)
Supplement: Supplementary file 1 [file metabolites-12-00029-s001.zip › Figure S3.pptx]

## Slide 1
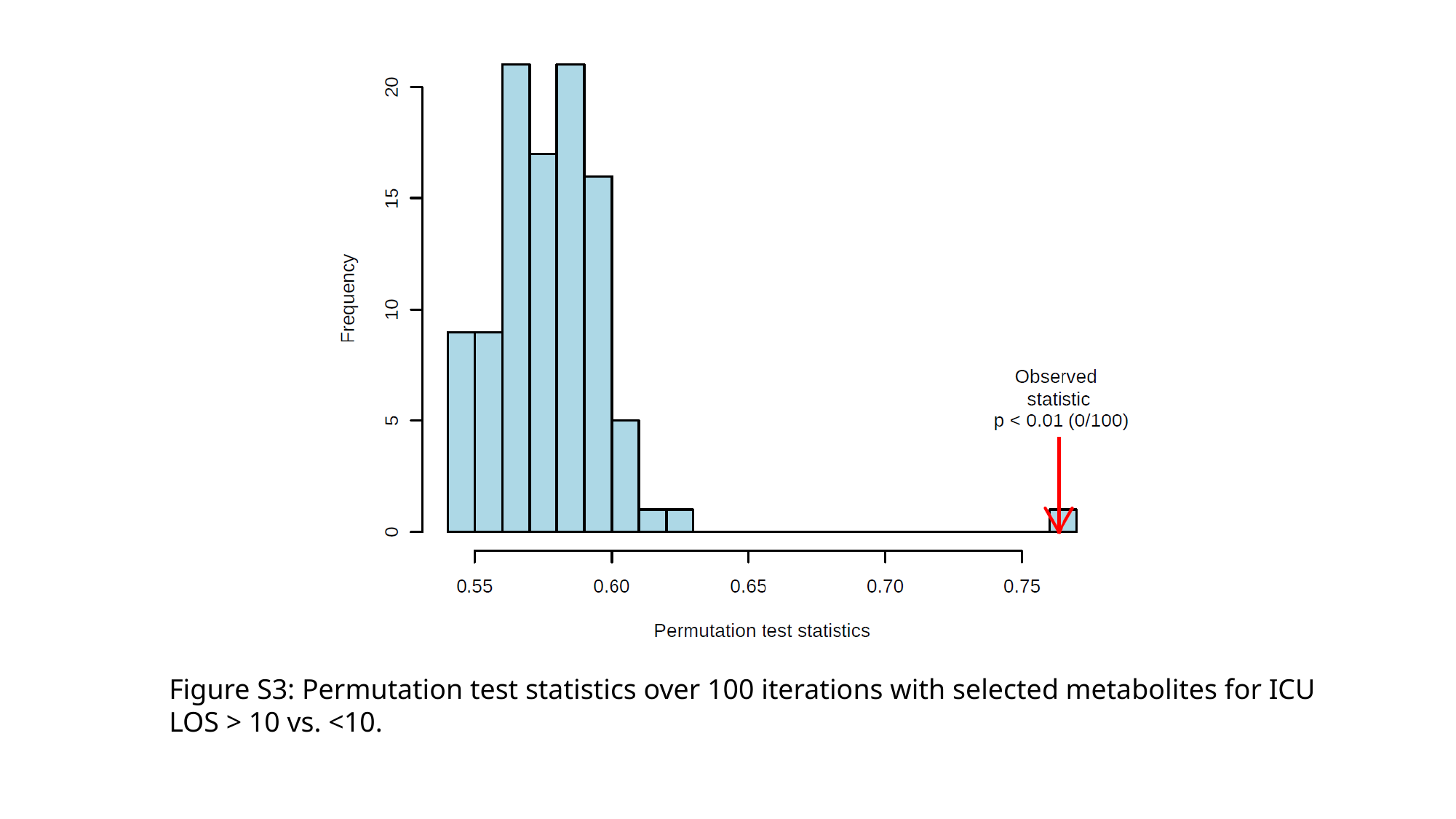

Figure S3: Permutation test statistics over 100 iterations with selected metabolites for ICU
LOS > 10 vs. <10.
