# Supplementary figures and images for: Integration of Metabolomic and Clinical Data Improves the Prediction of Intensive Care Unit Length of Stay Following Major Traumatic Injury
Source: Metabolites. 2021 Dec 31;12(1):29. doi: 10.3390/metabo12010029 (PMC8780653; doi:10.3390/metabo12010029)

## Slide 1
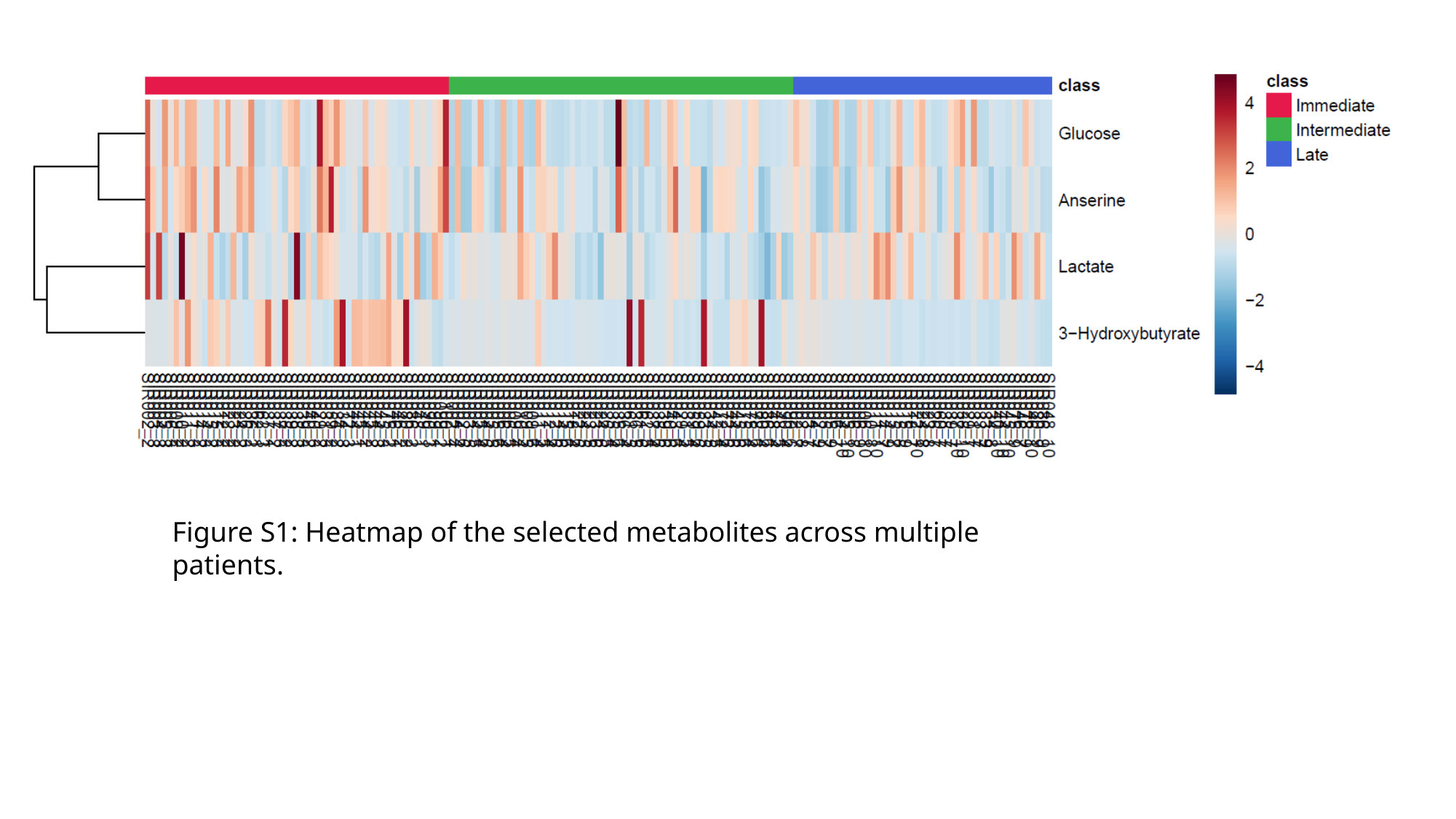

Figure S1: Heatmap of the selected metabolites across multiple patients.

Supplement: Supplementary file 1 [file metabolites-12-00029-s001.zip › Figure S1.pptx]

## Slide 1
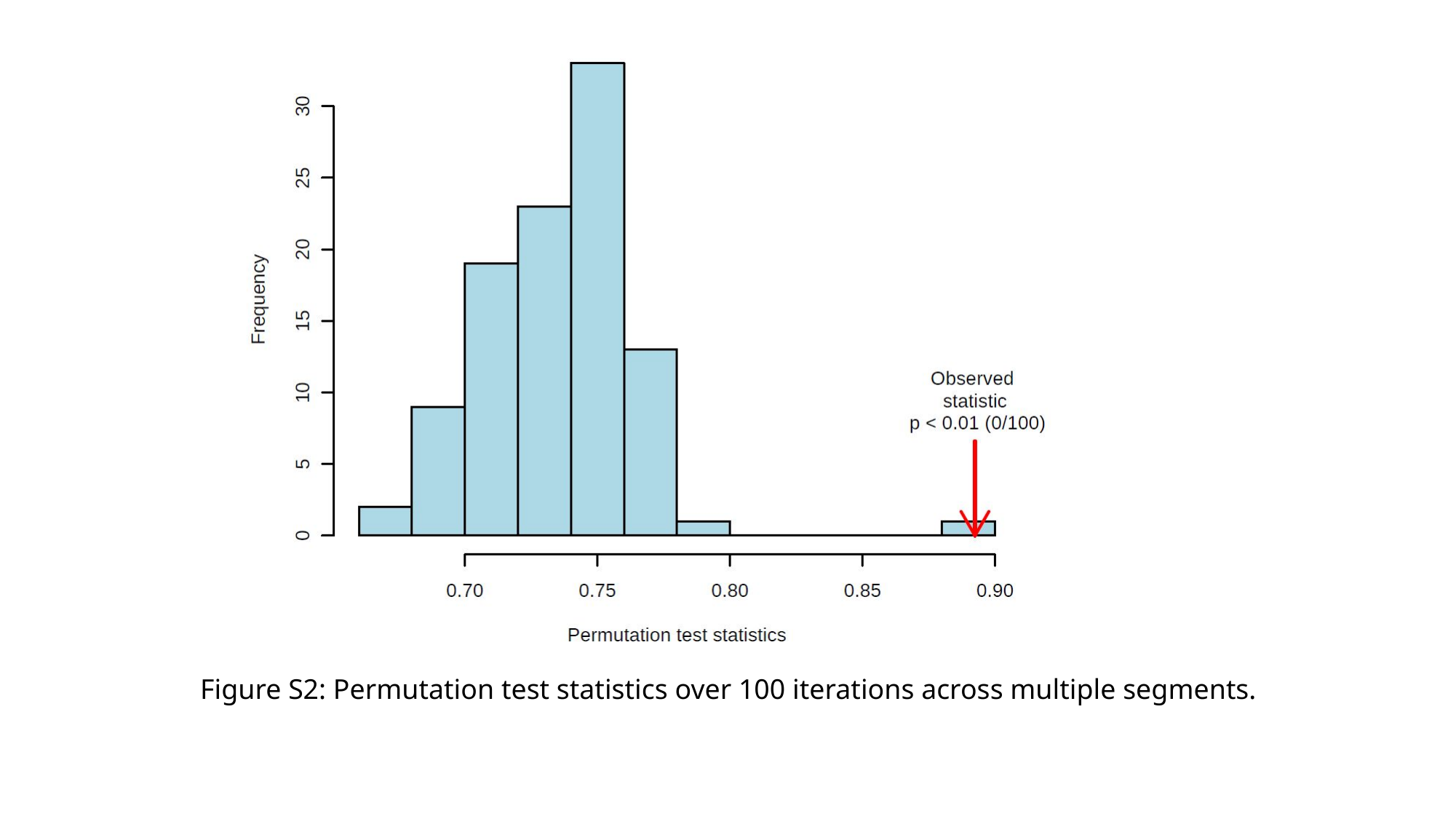

Figure S2: Permutation test statistics over 100 iterations across multiple segments.

Supplement: Supplementary file 1 [file metabolites-12-00029-s001.zip › Figure S2.pptx]
